# You’ll know when you’re ready: a qualitative study exploring how patients decide when the time is right for joint replacement surgery

**DOI:** 10.1186/1472-6963-14-454

**Published:** 2014-10-02

**Authors:** Barbara L Conner-Spady, Deborah A Marshall, Gillian A Hawker, Eric Bohm, Michael J Dunbar, Cy Frank, Tom W Noseworthy

**Affiliations:** Department of Community Health Sciences, University of Calgary, 3280 Hospital Drive NW, Calgary, AB T2N 4Z6 Canada; University of Toronto, 76 Grenville Street, Toronto, ON M5S 1B2 Canada; Department of Medicine, Women’s College Hospital, 76 Grenville Street, Toronto, ON M5S 1B2 Canada; Concordia Hip and Knee Institute, 310-1155 Concordia Avenue, Winnipeg, MB R2K 2M9 Canada; Department of Orthopaedic Surgery, Dalhousie University, Halifax, NS B3H 4R2 Canada; Faculty of Medicine, University of Calgary, 3280 Hospital Drive NW, Calgary, AB T2N 4Z6 Canada

**Keywords:** Decision making, Readiness, Total joint replacement, Qualitative research, Osteoarthritis

## Abstract

**Background:**

While some studies have identified patient readiness as a key component in their decision whether to have total joint replacement surgery (TJR), none have examined how patients determine their readiness for surgery. The study purpose was to explore the concept of patient readiness and describe the factors patients consider when assessing their readiness for TJR.

**Methods:**

Nine focus groups (4 pre-surgery, 5 post-surgery) were held in four Canadian cities. Participants had been either referred to or seen by an orthopaedic surgeon for TJR or had undergone TJR. The method of analysis was qualitative thematic analysis.

**Results:**

There were 65 participants, 66% female and 34% male, 80% urban, with an average age of 65 years (SD 10). Readiness reflected both the surgeon’s advice that the patient was clinically ready for surgery and the patient’s feeling that they were both mentally and physically ready for surgery. Mental readiness was described as an internal state or feeling of being ready or prepared while physical readiness was described as being physically fit and in good shape for surgery. Factors associated with readiness included: 1) pain: its severity, the ability to cope with it, and how it affected their quality of life; 2) mental preparation; 3) physical preparation; 4) the optimal timing of surgery, including age, anticipated rate of deterioration, prosthesis lifespan and the length of the waiting list.

**Conclusions:**

Patient readiness should be assessed prior to TJR. By assessing patient readiness, health professionals can elucidate and deal with concerns and fears, understand and calibrate expectations, assess coping strategies, and use this information to help determine optimal timing, both before and after the surgical consultation.

## Background

Total joint replacement (TJR) is highly effective in the management of advanced hip and knee osteoarthritis (OA) when non-surgical therapies fail. The elective nature of TJR creates an opportunity for an active discussion between the patient and the health care provider on the timing and appropriateness of surgery [1, 2]. A recent study of orthopaedic surgeons concluded that indications for total hip replacement must include an understanding of factors affecting a patient’s willingness to undergo surgery [3]. The consultation is central. It is where risks and benefits are discussed, trust established, and decisions made [4–6]. The patient and surgeon together must decide if the benefits of surgery outweigh the risks and the possibility of needing revision surgeries [4]. If all goes well, patient satisfaction – a major objective of the surgery – will be met [7–9]. However, 7% to 30% of patients report little or no improvement or are dissatisfied with the surgical result [10–15]. Dissatisfaction has been linked to pre-surgical pain [16], poorer mental health [13, 15], poorer outcomes [10, 14], complications requiring admission [16], and unmet expectations [7, 9, 16, 17], but results are inconclusive; many studies showed that pre-surgery variables have a minimal influence on post-surgery satisfaction [10, 18, 19]. Other reasons why a patient may not be satisfied with TJR are that the patient may not have been an appropriate candidate for surgery [20] or they may not have been ready for surgery [21].

There are no widely accepted guidelines on the optimal timing of surgery [22]. This decision is made by both the physician and the patient and considers various psychological, social, and other issues in addition to pain, disability, and x-ray changes [3, 23]. Delaying surgery could lead to worse surgical outcomes [24–26] while a major concern of performing surgery earlier, particularly on younger active patients, is long-term survival of the prosthesis [22, 27].

From the perspective of the patient, readiness for surgery is a key component in their decision whether to have hip or knee replacement surgery and is a potentially important factor in determining satisfaction with surgical outcomes [21]. In a study on patients’ perspectives of appropriateness for TJR, patients stressed the importance of assessing readiness when considering suitability for surgery and in obtaining a good outcome [28]. The concept of readiness for TJR has been described as the time when no other alternatives would be viable [29] and being mentally prepared for surgery [28].

There are a number of theories on behavioral change and decision making that can be used to shed light on patient decision processes. Models of behavioral change include the transtheoretical model (TTM) [30], the theory of reasoned action [31], the theory of planned behavior [32] and readiness to change [33]. As applied to the healthcare field, these pertain mostly to studies of health behavior change [34] such as smoking cessation [35], increasing physical activity [36], cancer screening behavior [37], and to patient readiness and barriers to starting treatment [38]. Theories of decision making include decisional conflict [39] and models of shared decision making [40, 41]; these require effective physician-patient communication including discussion of patient expectations and an appreciation and understanding of patient perspectives [42]. While some studies have identified readiness as a factor in patient decision making for TJR [28, 29], none have explored how patients determine their readiness for surgery. The purpose of this paper is to explore the concept of patient readiness and describe the factors patients consider when assessing their readiness for TJR.

## Methods

We recruited nine focus groups (4 pre-surgery, 5 post-surgery) in four cities across Canada: Halifax, Toronto, Winnipeg, and Calgary. The number of focus groups was based on feasibility and attaining representation from both pre- and post-surgery participants at all four sites. The focus group collects qualitative data through group interaction on a defined topic [43]. The objective is to collect data in a social context in which participants can consider their own views in context with the views of others through a process of sharing perspectives and experiences.

Ethics approval was obtained from the research ethics boards of the Universities of Dalhousie, Toronto, Manitoba, and Calgary. Eligibility criteria for the pre-surgery group were patients with OA who a) had an orthopaedic surgical consultation and were eligible for primary TJR or b) had been referred to an orthopaedic surgeon for consideration of TJR. The post-surgery group had to have had a primary TJR approximately 12 months previously. In three centres consecutive eligible patients were identified through the surgical registry and patient lists in the orthopaedic clinics and were contacted by a clinic nurse or research personnel at each site. Patients expressing interest were contacted by a research manager who explained the study and mailed them the study information and consent form. In the fourth centre flyers were posted in the orthopaedic hospital clinic. Interested patients contacted the research manager. A list of individuals interested in participating and their relevant demographic information (sex, joint, location, pre- or post-surgery, contact information) was sent to the focus group recruiter who confirmed eligibility and scheduled them into a pre- or post- surgery focus group depending on their availability. For both pre- and post-surgery groups the recruiter attempted to obtain representation from males and females, hip and knee patients, and urban and rural locations. Informed written consent was obtained and participants’ anonymity and confidentiality were addressed.

The research team developed a semi-structured interview guide based on prior research [44–49]. The interview guide was reviewed by the focus group moderator who was independent from the research team and experienced in conducting focus groups. Each focus group lasted approximately 2 hours with a refreshment break mid-way. Patients were initially asked to remember when they first considered having joint replacement: what was important, how they made their decision regarding having surgery, what factors did they consider and what information sources did they use to help them? Two researchers observed each focus group and took notes. Debriefing sessions between the moderator and researchers took place at breaks in the sessions and between focus groups.

All focus groups were audio- and video-taped and transcribed verbatim. The primary researcher read the transcriptions and verified them against the recordings to ensure accuracy.

The data were analyzed by qualitative thematic analysis [50, 51]. NVivo 8^©^[52] was used to assist in managing the data. The primary researcher and a second analyst independently coded the data and identified key concepts and relationships between concepts. This process involved a detailed reading of the text, labeling segments that represented a concept, comparing the concepts against one another and grouping concepts into themes. We explored each theme in its context by examining the conditions under which it occurred, and looked for relationships between themes across the data set. Themes were compared and discussed, and data revisited until a consensus was reached on the final themes. Exemplars for each theme were identified. To the best of our knowledge our manuscript reporting adheres to the RATS guidelines (http://www.biomedcentral.com/authors/rats) for reporting qualitative data.

## Results

There were 65 participants, 66% female, 34% male, and 80% from urban centers with an average age of 65 years (SD 10, range 28 to 89 years). Twenty six participants were pre-surgery and 39 were post-surgery. Of the pre-surgery group, 5 participants were waiting to see a surgeon (pre-consult) and 21 had seen the surgeon (post-consult). Of these 21, 14 had decided to have surgery and 7 were undecided or deferring the decision.

Patients used the term ‘ready’ to describe their decision regarding surgery and also the process of preparing themselves for surgery. They described readiness in terms of both mental and physical readiness. Mental readiness was described as an internal state or feeling of being ready or prepared while physical readiness was described as being physically fit and in good shape for surgery. This ‘readiness’ reflected the patient’s feeling that they were mentally and physically ready for surgery and the surgeon’s advice that the patient was clinically ready for surgery.

*He said you’re physically ready to go but you’re not mentally prepared for the surgery. That’s what I’ve been doing the last few months, been preparing in my head … I was physically ready, but in my head, I thought, oh my god this is too big … I’m frightened. It’s the idea of being a patient. I’ve always been a caregiver, not the patient. It’s a total reversal for me. I’ve got two more books to read before I’m ready. (female, age 54, on wait list)*

### Factors patients used to determine readiness for surgery

Patients considered many different social, personal and clinical factors to decide when they were ready for surgery. These are grouped into four main themes: 1) pain: its severity, the ability to cope with it, and how it affects their quality of life; 2) mental preparation; 3) physical preparation; and 4) the optimal timing of surgery, including considerations of age, anticipated rate of deterioration, prosthesis lifespan, and the length of the waiting list.

### Severity of pain and the ability to cope with it and its effect on quality of life

The prime motivation to see a surgeon was pain, coping with it, and how it affected their quality of life. Patients who were ready to have surgery had reached a point where pain and disability broadly affected their everyday life. They felt that they had no choice. Pain medications no longer worked or caused side effects. Sometimes, other joints were becoming affected. Quality of life was diminished; usual and necessary daily living activities were hindered and sleep was impaired. Often independence was lost, and the increased burden fell on their spouse and family. Social life was diminished, and work often became impossible. Patients also described irritability, depression, the diminished self-image because of using a cane, and how pain left them looking or feeling old.

*The social aspect of how big the disability cuts you off. Also stigma to using a cane. Even if you’re young, it makes you look old. (female, age 63, post-consult, declined offer of surgery)*

Some considered surgery as the final option. Many had postponed the decision but ultimately reached the point where they felt they had exhausted all other treatment options.

*You’re ready. You are desperate. Your pain, your quality of life, it’s intolerable and you’re willing to do anything. You have no other avenue. (female, age 51, post-surgery)*

Undecided patients, or those postponing surgery felt they could still cope with the pain, or could *‘go longer if the pain doesn’t get any worse’*.

*I don’t feel that I’m suffering enough to undergo surgery … there’s a tipping point, beyond which I feel that, okay, I’m going to have to do something. (male, age 66, post-consult, candidate, not ready)*

### Mental preparation

Patients weighed the expected benefits of surgery against the perceived risks. Much of the expected benefit centered around pain relief and the activities that this relief enabled. Surgery would let them walk, have a bath, sleep well, work longer, return to active sports like golfing, cross country skiing, and riding a bike. Regaining their mobility also meant independence, resuming a social life, improving their relationships with spouse and family, and *‘leading the life that’s appropriate for your age’*.

*To be able to do what people do, to be able to come and go, to be with people, to go to work, you know, to travel, just to experience, get out in the garden in sunshine, go out in rain and not hurt because of the arthritis. Essentially independence, going on your own. (female, age 58, on wait list)*

Surgery was associated with uncertainty and the fears of facing an unknown and uncertain future, of anaesthesia, of infections, of being cut, having the wrong joint operated on, of dying, and of failure--that it might not work and you would be worse off.

*I think one of the fears that I have, maybe everyone does, is that there’s always the chance it’s not going to work … I’ve heard horror stories. (male, age 62, on wait list, ambivalent)*

Patients living alone worried about who would help them after surgery, their need to rely on others, and about being alone*.*

*What terrifies me also is that I live alone and I don’t have anyone to take care of me. Who’s going to take care of me? You’re out of service for a long time. (female, age 69, post-consult, undecided)*

Patients described their decision whether to undergo surgery as *‘weighing the odds’*, *‘taking a gamble’*, *‘taking a chance’,* and *‘a calculated risk’*. For many patients this decision process was lengthy.

*It didn’t happen all of a sudden, but going through the process, the cortisone and elimination of things, finally you go for an x-ray and your GP says you have deteriorated, how do you feel about that? Should we go in for a hip replacement? And then again, the relationship I have with my GP with that trust factor. I’ve gone through the alternatives … I have to seriously consider … coming to grips with it. It’s very hard to accept. It’s a gradual process of accepting, of working through the issue with your own mind, and then you finally come to the realization that look, I have to make a decision,… finally you just mull it through your mind and then finally look, there are no 100% guarantees but at the same time if I keep deteriorating at the rate I am, what do I have left? Do I want this kind of a life and worse? Or do I want to take the chance? I have to take the chance if I want to change my life. (female, age 58, on wait list)*

Individuals going ahead with surgery concluded that the benefits outweighed the risks. Undecided individuals still weighed the pros and cons. Some thought they might have surgery sometime, but now they wanted to try alternative therapies. Some on the waiting list remained ambivalent about having surgery. For others it was an easy decision.

*The surgeon assures me I can hike and cross country ski and everything except running afterwards so for me it was like a 2 second decision. (female, age 68, on wait list)*

Readiness for surgery included mental preparation to deal with their fears and to gain a sense of control. Patients who had decided on surgery described various strategies to help them prepare and conquer their fears. These included information seeking, planning for aftercare, emphasizing the positive aspects of surgery, minimizing the risks, and putting their trust in the surgeon. One person described readiness as *‘being totally informed what to expect’.* Typically, patients received information booklets from their surgeon’s office before their surgeon consultation. But even after the consultation, questions often remained and some patients desired to meet their surgeon again, to verify the information received and to answer follow-up questions.

*I’d like a proper consultation with the surgeon because a lot of times, they’re so busy and they talk to you fast, and sometimes you think, I haven’t got the whole picture. (female, age 54, post-consult)*

In addition to information provided by the surgeon, patients sought information from reading, from other patients who had similar surgery, from information sessions, and by looking online. Patients wanted to know about the procedure, its risks, aftercare, and what outcomes to expect. These activities gave patients a sense of control and helped them plan for aftercare and anticipate a better quality of life.

*It’s really hard to internalize how rare those complications are because you keep saying to yourself, yes, I know it’s a long shot, but it did happen to someone, and so you have to keep reading to fight back some of that stuff and so it’s a teeter totter in terms of yes, you can hear a hundred good stories, but that one stays there. So it’s that fighting constantly internally that yes, it’s wonderful, but there is risk … Another thing that’s helped me a lot to decide was the session they had … sort of seminar, was seeing the diagrams, seeing the appliances that would be installed and actually understanding the process. I need to have a sense of control that comes from knowledge of the process and that really helped me. (female, age 67, on wait list)*

Other patients minimized the risks, tried to avoid stress, and emphasized the benefits of surgery.

*I don’t even remember if the doctor said there was a risk of an unfavourable result. I remember him saying there is a risk of infection and of a blood clot but I think I was just so ready for the surgery that I may not have heard other things and when I went in for the surgery I just went in thinking “I’m going to have my life back.” … I wouldn’t let myself think about what could go wrong. (female, age 55, post-surgery)*

Patients weighed the situation and experiences of others when making their own decision. They observed their outcomes, their hospital experience, and their post-surgery and recovery experiences.

*I weighed their situation as to my situation, their way of life to mine, you know, a whole bunch of things. Thinking well, mine won't be that bad because I'm not this, and mine won't be that good because I'm not this. (female, age 57, on wait list)*

Patients deciding to have surgery mostly noticed ‘success’ stories and noted what factors contributed to successful outcomes, such as a positive attitude and a determination to resume activities following surgery. One patient described it as a process of filtering. Talking to others with successful outcomes gave patients hope, especially if they had the same surgeon.

*I noticed their attitude. I think that makes a tremendous difference … The ones who were very positive and determined to get well and do whatever it was they wanted to do again, they got well and resumed their activities. (female, age 81, on wait list)*

For individuals who knew patients who had poorer outcomes, they would rationalize that other factors played a part, for example, the individual didn’t exercise before or after surgery.

*So a lot of times I think when you hear these reports where it did not go as forecast, all rosy, we need to do a bit more digging and ask what did you do to contribute to your demise with the operation. (male, age 60, on wait list)*

The face-to-face meeting with the surgeon was important in establishing a trust relationship, allaying fears, and enabling the patient to feel confident in their decision. Most patients who had met with a surgeon had a trusting relationship with their surgeon.

*That you’re going to use all your skills and knowledge to the best of your ability to help me as much as you possibly can. (female, age 59, pre-consult)*

### Physical preparation

Patients also described readiness in terms of being physically fit and in good shape for surgery. They took steps, such as weight loss and physical exercise, to make their rehabilitation less strenuous and improve their chances of a good surgical outcome. Some felt that if they lost weight, maybe they wouldn’t need surgery.

*I knew I wanted to lose weight and, of course he wanted me to lose weight too because that would make the best possible surgery, but I also looked into okay, what other things can I do? I actually do some weights to keep my upper body strong so I can use my walker and not hurt my back, which in essence will keep me mobile. And trying to prepare for the surgery physically and mentally. (female, age 57, on wait list)*

### Optimal timing for surgery

Readiness involved determining the optimal time for surgery. Patients considered their age, their rate of joint deterioration, the lifespan of the prosthesis, and the anticipated wait time. Age was considered primarily in terms of the lifespan of the prosthesis and how many revisions a patient might have. Some patients were told they were too young for surgery or had received conflicting information as to the minimum age for surgery.

*I went to see one surgeon at that point and he advised that I wait until I was at least 55 and I think I was 50 at the time and he didn’t want to do both knees at the same time. I went to see another surgeon and he said that his belief was quality of life is more important than chronological life. He asked me if I was at the end of my rope and I told him that basically I wanted my life back. He was quite open to doing both at the same time. (female, age 55, post-surgery)*

Others worried about what they would do in ten to fifteen years when they might need a revision. People also considered the years of living with a poor quality of life if nothing was done. Some patients in their 40s or 50s wanted surgery so they could enjoy 20 or 30 years of improved quality of life, while others wanted to wait in case something went wrong. Patients also weighed the consequences of waiting - fears of being in a wheelchair, or deterioration in their condition, and their ability to deal with the pain if their condition worsened.

*I still have a good number of working years to go and so it becomes a choice, I can gamble and take the chance that it will work out … or the dice goes another way. But either way, I would have deteriorated over time and by the time I’m retiring age I would have deteriorated so much that I would have been bedridden. If I don’t take a chance, my chances of my quality of life will deteriorate extremely - what do I have left then? I won’t make it through my working years, never mind my retirement years. Why do they want to hold off if you’re below 60? The appliance only lasts so long, but now with these new ceramic appliances it extends the life of it. Now, if I need the surgery at my age, okay. To me, quality of life is more important now than when I’m 70 or 80. Give me the chance now and then if we have to go through it again when I’m in my 80’s or 70’s, okay, then do it again, but why waste the good years when you still have them? (female, age 58, on wait list)*

Patients relied to varying degrees on their surgeon’s opinion regarding the right time for surgery. *‘When your doctor says you’re ready, you’re ready.’ (female, age 73, on wait list)* In some cases, the surgeon left the timing to the patient. *‘He has said when you’re ready, you’ll let me know.’ (female, age 69, post-consult, undecided)*.

The knowledge of long waiting times influenced the decision of some patients as to when to go on the wait list for surgery. Once patients agreed to have surgery, they knew the wait time was long.

*Unfortunately, by the time we feel that we need to have the surgery, then we're faced with a wait list. Or, a waiting time. So, we feel like our lives are on hold. (female, age 57, on wait list)*

Because they expected a lengthy wait, some patients agreed to surgery before they were ready and were worried that their condition would deteriorate.

*He said that you should probably think about getting it done, so I said okay because I knew I'd be waiting. I wasn't ready at the time. (female, age 62, on wait list)*

Some individuals on the waiting list expressed ambivalence about their decision regarding surgery while others, who went on the list preemptively, expressed dissatisfaction and regret that they had had the surgery.

*Mine was a preemptive thing. I didn’t want it to get worse and it wasn’t explained to me that it wouldn’t matter. If I had known the risks of postponing, which weren’t drastic, I would have postponed it. (female, age 73, post-surgery)*

*I was preemptive as well in a sense that I was afraid of going to the end of the line. My fear of waiting times was a major factor in my decision, probably the pre-eminent one…. if I didn’t do it now, when he said, “Are you ready?”, I thought I’d have to wait another 16 months and I can’t predict that I can hang on that long. (female, age 51, post-surgery)*

## Discussion

We explored the concept of patient readiness and how patients decided if and when they were ready to proceed. These considerations provide important input to the joint surgeon-patient decision regarding having TJR and also the optimal timing for the patient. We focused on the decision making process of patients who had sought a consultation with an orthopaedic surgeon and who were deciding or had decided to undergo surgery. This is in contrast to qualitative studies of OA patients in primary care practices who may have considered TJR [29], and to population studies of individuals unwilling to consider TJR despite potential eligibility [44, 45, 48]. We also asked patients who had undergone TJR to reflect on their decision making process.

Patient readiness emerged as a key concept in the decision to have joint replacement surgery. This concept involves two overlapping phases. The first refers to the steps a person goes through to decide if they will have surgery. The second refers to the preparation needed for the surgery before the surgery takes place. Both phases have mental and physical aspects. The biggest part of readiness is the mental preparation, addressing concerns around pain, quality of life, social isolation, assessing risks and benefits, facing fear and uncertainty, and if “the time is right” for surgery. Frankel et al. found that patients felt that better surgical outcomes were linked to a positive attitude, motivation, will power and feeling mentally prepared [28]. In addition to mental preparation, we found that patients who prepared physically felt that being in better shape before surgery would give them a better surgical outcome.

Similar to other findings [29, 53], patients decided on surgery when pain and disability broadly affected their everyday life; some saw surgery as the only option left after exhausting all other treatment options. We found that undecided patients were still weighing the pros and cons of surgery whereas most patients who decided to have surgery felt that the expected benefits outweighed the perceived risks. However, some patients who decided to have surgery and were on the surgical wait list remained ambivalent or uncertain about their decision. This uncertainty or decisional conflict could be due to lack of information about the alternatives and their consequences or to the emotional distress associated with making a choice involving risk and uncertain outcomes [39].

Addressing the fears of surgery and the uncertainty of their outcome appears to be the major hurdle many patients face when deciding on surgery. The fears associated with TJR were similar to those reported by others [29, 44, 53] but we found that patients deciding to have surgery used various strategies to cope with their fears. Approach and avoidance are common ways to cope with fear [54]. The purpose of an approach strategy is to gain control over the threat relevant aspects of the situation, whereas an avoidance strategy works to reduce the arousal associated with a threat. In our study, patients used both strategies to resolve their fears. Approach strategies included going to seminars, reading, using the internet, and observing others and asking them about their experiences. We found that patients who had decided to have surgery commonly referred to the positive outcomes they saw in others. Those who were uncertain referred to others with both positive and negative outcomes. In contrast, other studies have shown that individuals who were unwilling to consider TJR [44, 45] formed their fears and perceptions of surgery largely from the negative accounts of others and on lay sources that were often inaccurate or unreliable. Emphasizing the potential benefits of the surgery while minimizing the risks and putting their trust in their surgeon are coping strategies that reduce the anxiety of the threat.

Consistent with others [5, 45, 55, 56], many patients defer surgery for years before finally proceeding. Knowing the optimal time to have surgery was not always clear to patients and they did not always receive consistent information from surgeons. From the patients’ perspective, factors involved in timing included their quality of life, the rate of deterioration, their age, the lifespan of the prosthesis, and the anticipated length of waiting time. Patients with disabling arthritis in Ontario who were unwilling to consider TJR were less likely to have spoken with a physician about joint replacement, less likely to perceive their arthritis as severe, less likely to accept the potential risk of revision surgery, and did not necessarily consider themselves a candidate for TJR [45, 57, 58]. In contrast to individuals unwilling to consider TJR [44, 45, 48], our patient sample had sought orthopaedic consultation or had already decided on surgery. Therefore, the optimal timing of surgery became an important issue in their decision making. They weighed not only the expected benefits against perceived risks but also the more immediate benefits against the rate of deterioration and the potential need for revision surgery in the future. Although age was a consideration in the timing of surgery for our patients, they did not view their arthritis as a normal part of aging, a common perception amongst individuals unwilling to consider TJR [45, 48]. This was likely because patients seeking surgeon consultation were further along the continuum of decision making and had accepted that TJR was a realistic option to deal with their OA pain at some time in their future.

Much as the patient must assess their need for surgery, the desired timing, and appropriate circumstances, surgeons must also assess need, timing, and appropriateness. Surgeons both differ and are inconsistent in their opinions about the most appropriate candidate for TJR [3, 59]. In a Canadian survey of orthopaedic surgeons, 86% were reluctant to perform TKR (knee) on patients under 55 years [59]. Knowing the optimal timing for surgery is important for both patients and surgeons, as compromised surgical outcomes are more likely when surgery is too early or too late [3, 60]. Thus, appropriateness pertains not only to the right procedure for the right person but also at the right time [61]. Surgeons need to know the optimum window of opportunity for each patient to achieve the maximum benefit [60]. Delaying surgery may lead to worse outcomes. Conversely, if patients go on the waiting list prematurely, due to long wait times and a fear of losing their place on the waiting list, they may be dissatisfied, particularly if their outcomes are not as expected.

The important fact is that, to varying degrees, both patient and surgeon are involved in the decision-making process. Surgeon-patient agreement on the patients’ health status and the most appropriate treatment is a prerequisite for informed and shared decision making and may contribute to better outcomes [4]. This shared decision making is seen as a central feature in patient – centered care [40, 62].

Decision aids are useful tools to facilitate this process and have been developed to support patient decision making and treatment choices for hip and knee OA [63–65]. They are designed to help patients make an informed choice between two or more treatment options [66–69]. They help patients to understand relevant information, to clarify their attitudes towards potential benefits and harms, and to aid communication. At present, they are not routinely used in orthopaedic surgery [20, 66].

A recent study showed that although orthopaedic surgeons frequently discussed the nature of the decision, alternative treatments, and risks and benefits with the patient, they rarely discussed the patient’s role in decision making or assessed the patient’s understanding [70]. More active attention to this in the patient-surgeon consultation may increase patient-surgeon consensus on beliefs about the need for and risks and benefits of TJR [4]. The patient and surgeon together must decide if the benefits of surgery outweigh the risks of needing multiple revision surgeries. The failure of clinicians to understand and manage expectations and acknowledge patient preferences, and the lack of congruence between patient and clinician perceptions of the patient's need can lead to patients feeling anxious and discontented [5].

A study limitation is although the sample was drawn from four orthopaedic centres across Canada, results may not be transferable to patients across Canada referred for similar surgery. However, similar themes emerged across the nine focus groups. We did not collect demographic data other than age and gender, thus, could not report on other patient characteristics such as ethnicity. We did not analyze the data based on gender but this would be an important aspect to explore in future research. Although we stratified patients by pre- or post- surgery, we had too few patients in the pre-consult group at each site to stratify patients by pre- or post- consult. While we attempted to recruit both satisfied and dissatisfied patients for the post-surgery groups, it was more difficult to find dissatisfied patients. Another limitation is that our data were not compared with complementary methods to examine various dimensions of the underlying phenomena; this could have been done via interviews or focus groups with patients’ families or physicians and may have helped to provide more insight into readiness for surgery. To strengthen the trustworthiness of the findings and reduce researcher bias, an independent focus group moderator was used, debriefing sessions took place between the moderator and researchers, and two researchers independently coded and analyzed the data.

Further exploration of the relationship between patient readiness for surgery and satisfaction with surgery would be valuable. We found only one study that assessed the effect of patient readiness or preparedness on post-surgery satisfaction. Patients who felt prepared prior to reconstructive pelvic surgery reported greater post-surgery satisfaction and greater improvement in quality of life [21].

## Conclusions

In conclusion, this paper focuses on the concept of patient readiness and what it means to patients in the context of patient decision making for TJR. By assessing patient readiness before TJR, health professionals can elucidate and deal with concerns and fears, understand and calibrate expectations, assess coping strategies, and use this information to help determine optimal timing.
